# The consciousness-first commitment of integrated information theory

**DOI:** 10.1093/nc/niag074

**Published:** 2026-11-02

**Authors:** Jeremiah Hendren

**Affiliations:** Graduate School Language & Literature, Ludwig Maximilian University of Munich, Geschwister-Scholl-Platz 1, Bavaria, 80539 Munich, Germany; Department of Psychiatry, University of Wisconsin–Madison, 6001 Research Park Blvd, Madison, WI 53719, United States

**Keywords:** consciousness, integrated information theory, explanatory identity

## Abstract

Integrated information theory (IIT) accounts for consciousness through its *explanatory identity*: an experience (the *explanandum*) is identical to a causal structure (or *Φ*-structure, the *explanans*). In other identities in science (e.g*.* ‘water is H_2_O’), the power of the explanans (H_2_O) comes from the presumption that it somehow exists more fundamentally. Yet when our explanandum is experience itself, as in IIT, we must recalibrate the norms of explanation to the fact that experience exists immediately and irrefutably, while whatever we may posit about the world beyond experience is always an inference within experience.

In this issue, Signorelli *et al.* (2026) offer various criticisms of integrated information theory (IIT), the most consequential of which concerns the theory’s *explanatory identity*. Before addressing their critique, I will briefly explain this core conjecture of IIT.

IIT aims to account for consciousness in objective terms (IIT Wiki/Overview [Hendren *et al*. 2024]; Albantakis *et al*. 2023).^1^ The first step is to use introspection to identify the properties of experience (Fig. 1, left), some of which are essential (e.g*.* all experiences are integrated) and others ‘accidental’ (e.g*.* some experiences include the color red, others do not). IIT then aims to account for these phenomenal properties in terms of causal properties of a substrate of units in a state—for example, a group of neurons (Fig. 1, bottom right). The reasoning process that follows culminates in the explanatory identity: every property of experience corresponds, one to one, to a property of the causal structure (or Φ*-structure*) ‘unfolded’ from the substrate (Fig. 1, top right; IIT Wiki/Identity).^2^ The identity is explanatory in the sense that the causal properties (the *explanans*) offer a physical account of the phenomenal properties (the *explanandum*).

**Figure 1:**
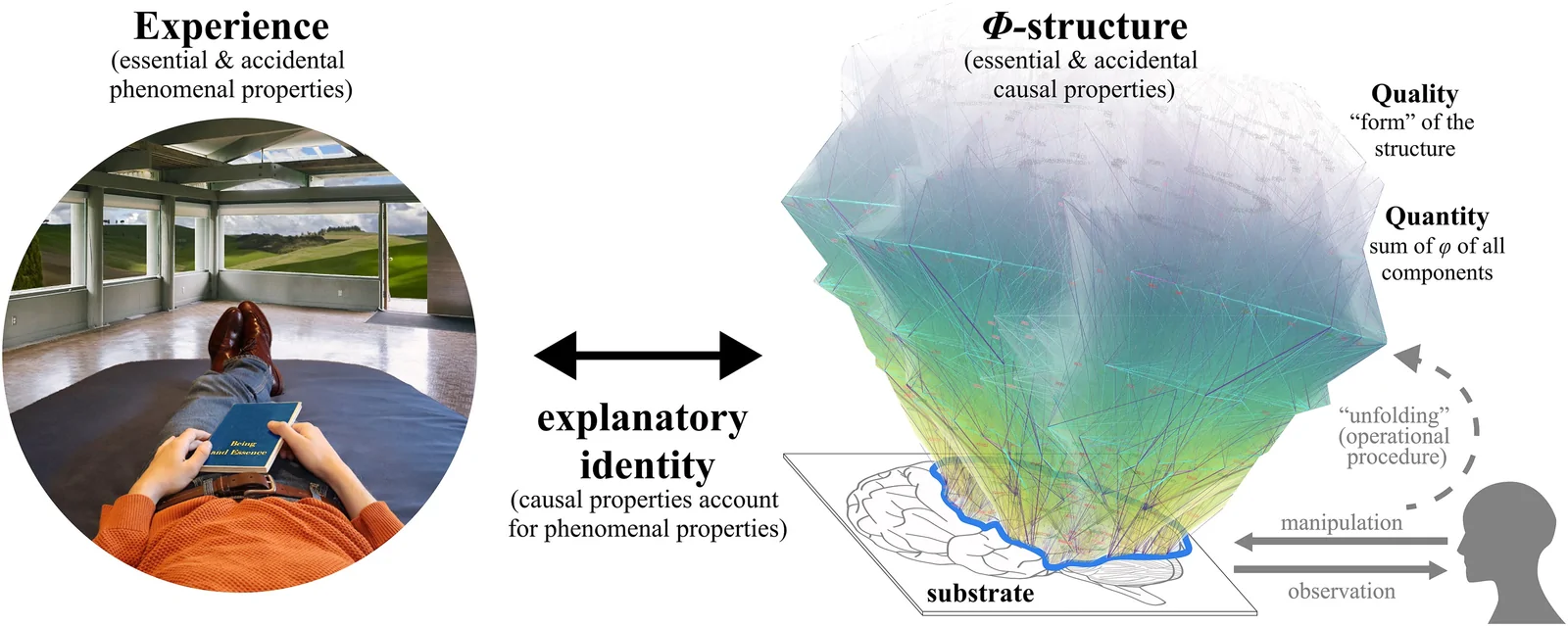
The explanatory identity of IIT. (Adapted with permission from Hendren *et al.* 2024.)

Signorelli *et al*. (2026) argue that there is ‘a problematic dissociation between the ontological and explanatory levels of discourse […]. IIT’s explanation takes the ontologically primary as explanatorily secondary’ (p. 7). They illustrate this with their ‘impossible tribar’ figure and appeal to canonical examples (p. 8):

…explanatory identities explain[] the relevant properties as described in [a] familiar, epistemically primary sense (e.g. water as transparent), by inferred, theoretical, or indirectly observed properties, which are, in that sense, epistemically secondary (e.g. *H_2_O* molecules). This explanation works, however, because the epistemically secondary, the explanans (e.g. *H_2_O*) is both ontologically and explanatorily primary: it reveals the true nature of the phenomenon and explains it.

The authors see IIT’s explanatory identity as being ‘contrary to seemingly all the best-known explanatory identities in the history of science’ (p. 8) because the explanandum remains ontologically primary throughout. This is true. Why? Because the explanandum is subjectivity itself—something genuinely new under the sun of scientific explanation.

It may be helpful to set aside the philosophy-of-science lens and think instead in everyday terms of perspectives. IIT aims to account for first-person phenomena in third-person terms. The main first-person tool is introspection—‘looking inside’ our experience. The main third-person tool is perturbing and observing things ‘out there’—for example, a brain (Fig. 1, bottom right). IIT keeps these perspectives (and tools) separate. We don the first-person perspective to articulate what we aim to explain—that is, the properties of experience, which we know ‘immediately’ or ‘by direct acquaintance’ (IIT Wiki/Foundations). We don the third-person perspective to do the explaining, but without reducing one perspective to the other. In IIT, the explanans does not reveal the ‘true nature’ of the explanandum; it rather offers a new view on it.^3^ After all, the true nature of experience is and always will be ‘what it is like to be’ us (Nagel 1974).

All other identities in science explain one third-person ‘thing’ in terms of another (e.g. water in terms of molecular theory). As such, it may make sense to treat the explanans as ‘both ontologically and explanatorily primary.’ But since subjectivity is a categorically different explanandum, it is reasonable—in fact, essential—that we recalibrate the norms of explanation to this unique scientific endeavor. The reason is Descartes’s timeless skeptical impulse, often mentioned but rarely heeded (Sarkar 2003): whatever may be true of the outer world, my experience exists—immediately and irrefutably.^4^

Figure 2 contrasts typical reductive identities with IIT’s explanatory identity. On the left side, water reduces to H_2_O in an explanatory sense, which many also interpret as an ontological reduction (though ontology is usually left implicit in science). Yet a complete account cannot avoid the fact that this identity exists first and foremost within experience, as indicated by mimicking Mach’s ‘View from the left eye’^5^: who knows whether water is really ‘nothing but’ H_2_O, but in my experience this helps explain its properties. For most pursuits in science, it appears harmless to ignore the primacy of experience, but not when experience itself becomes our explanandum.^6^ This is illustrated by the right side of Fig. 2, presenting IIT’s explanatory identity from a fresh perspective: a *Φ*-structure unfolded from a portion of my brain can account for my experience in physical terms.^7^ But must this account negate the primacy of experience? No—my experience cannot be erased.

**Figure 2:**
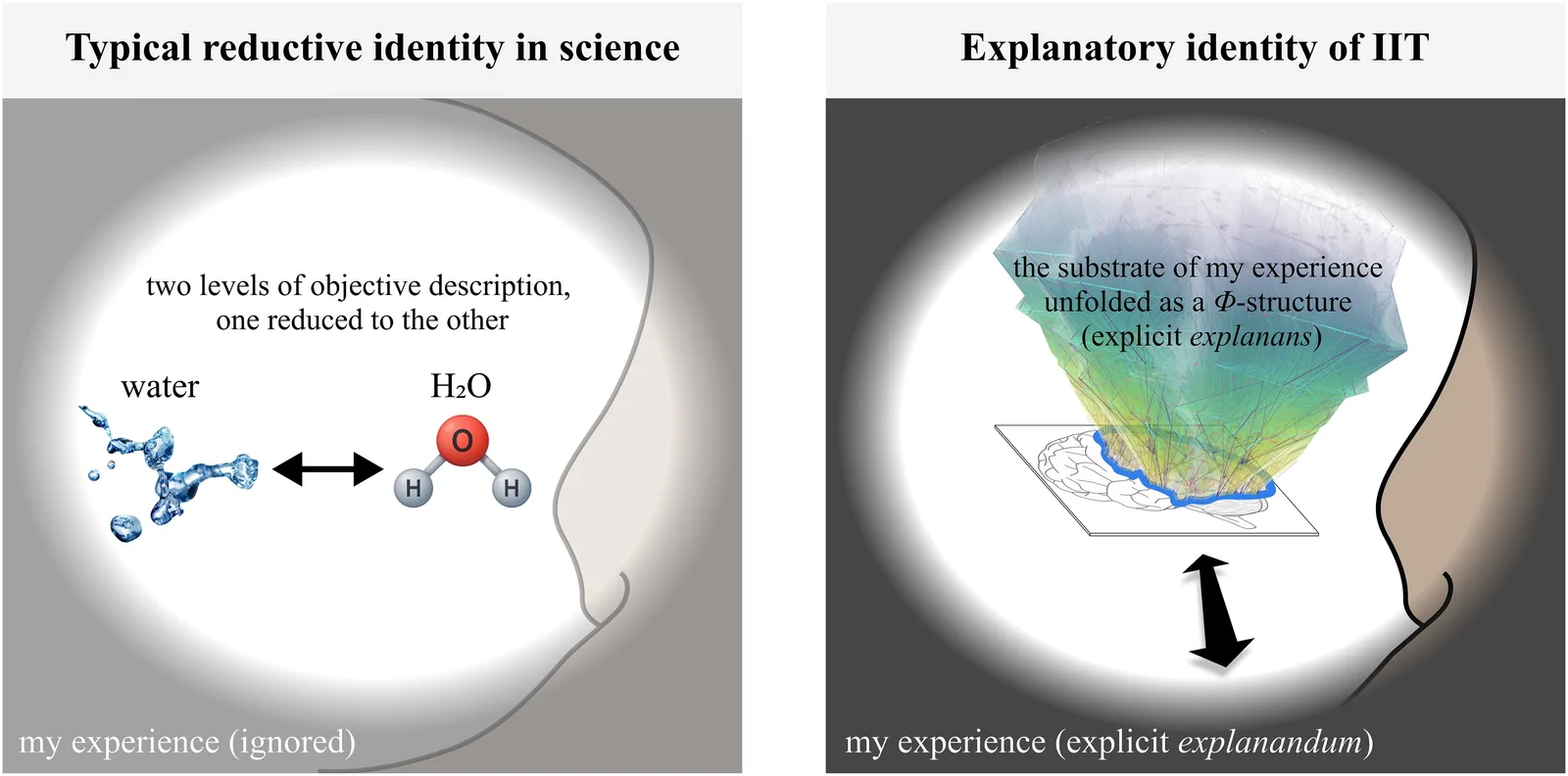
The first-person perspective in typical reductive identities versus IIT’s explanatory identity.

IIT upholds the primacy of experience from start to finish. Epistemologically, my experience is primary since trying to doubt it ends up confirming it (as demonstrated by Descartes’s *cogito*). Ontologically, my experience is also primary in that its existence neither demands nor can have any proof other than itself. That said, this does not make the *Φ*-structure corresponding to my experience ontologically ‘secondary’—such framing risks dualist overtones. We might rather call the *Φ*-structure equally primary—as a third-person view of first-person phenomena. What we could call ontologically ‘secondary’ in IIT is the substrate *per se* (Tononi and Boly 2025). My brain, for example, appears to exist as a unitary ‘thing’ for an outside observer—it exists *extrinsically*, in IIT’s terms (Fig. 1, bottom right). But only by manipulating and observing the substrate can the observer unfold its causal powers in full and determine which portion of it exists *intrinsically*, for itself, as a *Φ*-structure—a structure of causes and effects.

It is worth pausing to note that the criticism of Signorelli *et al*. (2026) would be valid if IIT were an emergentist or functionalist theory, as it is often misconstrued to be. In other words, the theory would be in trouble if it argued that experience ‘arises from’ the causal properties of a substrate, or that experience is a ‘higher-level function’ of that substrate. In such frameworks, the substrate is indeed ‘both ontologically and explanatorily primary.’ But consciousness is a question of *being*, not *doing* (Tononi and Boly 2025). Since experience *is*—in an actual sense, right here and now—it cannot be identical to something the physical substrate *does* but only to what it *is* intrinsically—again, a *Φ*-structure.

Even though IIT’s explanans (*Φ*-structure) does not reveal the ‘true nature’ of the explanandum (experience), it may still yield insights that we cannot glean from the first-person perspective alone. The first step, however, is to demonstrate the construct validity of IIT’s explanatory identity. This is currently being pursued by accounting for experiences that are partly penetrable to introspection—namely, space, time, and objects (IIT Wiki/Contents). If the identity can be validated for such experiences, this warrants future work probing the identity in the ‘other direction’—from our knowledge of a neural substrate to phenomenology.^8^ We might use knowledge about, say, neural circuits responsible for color or pain to shed light on these phenomenological properties—notorious for being difficult to decompose through introspection. Would this require the physical explanation to be ontologically more fundamental than the experience itself? No—but it would indeed ‘modify our understanding/concept of the target phenomenon’ (p. 8), which Signorelli *et al*. (2026) rightly flag as an important role of the explanans.

I have emphasized the split between first- and third-person perspectives in IIT. In closing, What about the second-person? This perspective is what enables intersubjective agreement—for example, on whether my first-person perspective is similar to that of others (e.g*.*  Kawakita *et al*. 2025). On this point, I agree with Signorelli *et al*. (2026) that future IIT work could profit from intersubjective phenomenological methodologies to complement introspective ones.

## Data Availability

No new data were generated or analyzed in support of this research.

## Footnotes

1
*Consciousness*, *experience*, and *subjectivity* are roughly synonymous in IIT.

2 ‘Unfolding’ refers to the computational operations applied to the substrate following the postulates of IIT (IIT Wiki/Unfolding).

3 This is not to downplay the importance of this new perspective. In fact, the hope is that it will lead not only to ground-breaking clinical applications but to a unified scientific view of nature and our place within it (Tononi and Boly 2025).

4 This paraphrase captures the thrust of Descartes’s argument in the language of IIT’s ‘0th axiom’ (IIT Wiki/Foundations). This approach resonates somewhat with Sarkar (2003), who offers a thorough discussion of the *cogito* not as an ‘argument’ but as an ‘intuition’ or an ‘experiment.’

5 Fittingly, Mach used this drawing to support a similar argument.

6 This core point resonates with, for example, Frank *et al.* (2024) and Goff (2017).

7 This depiction is awkward since it shows ‘me’ looking at the substrate of my own experience. It is merely a rhetorical device to stress the primacy of experience.

8 Note that this explanatory strategy also resonates with Varela’s (1996) proposal of ‘neurophenomenology.’
